# De-escalation strategies in early breast cancer: implications of sentinel lymph node biopsy omission for adjuvant radiotherapy

**DOI:** 10.1007/s00066-025-02391-2

**Published:** 2025-04-16

**Authors:** K. J. Borm, J. Hörner-Rieber, N. M. Duma, W. Budach, M. D. Piroth, D. Krug, Breast Cancer Expert Panel of the German Society of Radiation Oncology DEGRO

**Affiliations:** 1 TUM School of Medicine, Department of Radiation Oncology, Technical University of Munich, Ismaningerstraße 22, 81675 Munich, Germany; 2 Department of Radiation Oncology, University Hospital Düsseldorf, Düsseldorf, Germany; 3 Department of Radiation Oncology, Helios Clinics of Schwerin-University Campus of MSH Medical School Hamburg, Schwerin, Germany; 4 Department for Human Medicine, MSH Medical School Hamburg, Hamburg, Germany; 5 Department of Radiation Oncology, HELIOS University Hospital Wuppertal, Witten/Herdecke University, Heusnerstraße 40, 42283 Wuppertal, Germany; 6 Department of Radiotherapy and Radiation Oncology, University Medical Center Hamburg-Eppendorf, Hamburg, Germany

Recently, two studies focusing on the omission of sentinel lymph node biopsy (SLNB) in patients with clinically node negative breast cancer were published: the prospective non-inferiority SOUND trial [1] included women of all ages with invasive breast cancer up to 2 cm with preoperative axillary ultrasonography showing no lymph node involvement and with a plan to undergo breast-conserving surgery and radiotherapy. The primary endpoint of the study was distant disease–free survival (DDFS). Among 1405 women with a median age of 60 years and a median follow-up of 5.8 years, distant metastasis-free survival was non-inferior for patients without axillary surgery compared to those who underwent SLNB. The number of locoregional relapses was low in both groups (1.7% in the SLNB group vs. 1.6% in the no-SLNB group).

The INSEMA trial [2] included breast cancer patients with a tumor size of ≤ 5 cm and clinically node-negative disease undergoing breast-conserving surgery. Patients were randomized 4:1 to SLNB surgery vs. no axillary surgery. The primary endpoint was invasive disease-free survival (iDFS). The median age was 62 years. After a median follow-up of 6.1 years, the 5‑year iDFS was 91.9% for no axillary surgery vs. 91.7% for SLNB, showing non-inferiority for the elimination of SLNB. Locoregional recurrences were 1.4% vs. 1.9%, and axillary recurrences were 0.3% vs. 1.0%. Omission of SLNB led to a lower incidence of lymphedema, greater arm mobility, and less pain with arm/shoulder movement and was further associated with superior quality of life (QoL) [3].

These two trials are practice changing for patients with early-stage breast cancer, given the excellent results despite the elimination of SLNB. Of note, approximately 95% of the women in both trials had hormone receptor (HR)-positive, human epidermal growth factor receptor 2 (HER2)-negative breast cancer; approximately 90% were postmenopausal; and approximately 90% of the INSEMA and all SOUND patients had pT1 tumors, despite the broad inclusion criteria. While there is an ongoing discussion regarding the optimal selection criteria for SLNB omission and the potential implications for systemic therapy, the consequences for adjuvant radiotherapy as an important part of breast-conserving therapy also urgently need to be addressed.

In both trials, radiotherapy was a crucial part of breast-conserving therapy, with 97.6% of patients in the SOUND trial receiving radiotherapy and 100% in the evaluated cohort of the INSEMA trial. The fact that 8.7% and 11.6% of patients in the SLNB arms, respectively, had macrometastases and that axillary lymph node recurrences still only occurred in 0.4% and 1% in the no-SLNB arms, respectively, with a follow-up of approximately 5 years indicates that the incidental dose coverage of the axillary region may have contributed to eradicating residual axillary disease.

The discussion regarding the effect of incidental lymph node irradiation during adjuvant radiotherapy in breast cancer is not new, as the effect of incidental dose coverage was comprehensively discussed after publication of the ACOSOG Z0011 data, in which no difference between ALND and observation following positive SLNB was observed for patients with tumors < 5 cm who received whole-breast irradiation, even though 27.3% of patients in the ALND arm had macrometastases (> 2 mm) in non-sentinel nodes [4–7].

The incidental dose distribution in the axillary levels varies widely among different patients. Several factors that have an impact on the incidental dose in the axillary lymph nodes have been investigated, including the upper field border and treatment technique, such as intensity-modulated radiotherapy (IMRT) and volumetric intensity modulated arc therapy (VMAT) vs. three-dimensional (3D) conformal radiotherapy (3D CRT) and irradiation in deep-inspiration breath hold (DIBH), as well as individual patient anatomy [8–13]. The clinical implications of these differences remain largely unclear.

The discussion regarding incidental irradiation to the axilla in cases of SLNB omission is of importance, as the patient collective that is most suitable for de-escalation of SLNB according to the authors of the trials—namely pT1, G1–2, cN0, HR positive, HER2 negative, and > 50 years—are also candidates for partial-breast irradiation and, for some of the elderly patients, even omission of RT could be discussed. Theoretically, even escalation of treatment with regional nodal irradiation of the supra-/infra-clavicular and internal mammary regions may be an option for a subgroup of patients with undetected involved lymph nodes. Since the overall prognosis of patients in these trials was excellent and regional nodal irradiation would mostly be considered for higher-risk patients not fulfilling the abovementioned criteria, this will not be further discussed in this article.

Thus, two questions need to be addressed: should all patients undergo adjuvant radiotherapy in the context of de-escalation of axillary surgery and can these patients undergo partial-breast irradiation?

## Omitting axillary surgery: implications for whole-breast irradiation

Several studies have investigated the omission of radiotherapy in low-risk early breast cancer patients. Even though recently published single-arm studies such as LUMINA and IDEA [14, 15] have found very low rates of local recurrences after 5 years in highly selected patients with both low clinical and genomic risk treated with endocrine therapy without radiotherapy, randomized trials (CALGB 9343, PRIME II) comparing endocrine therapy (ET) alone versus ET + RT with longer follow-up (≥ 10 years) showed significant differences in locoregional recurrence rates: 2% and 1%, respectively, versus 10% without radiotherapy [16, 17]. Furthermore, in both studies (CALGB 9343 and PRIME II), a doubling of local recurrences after 10 years vs. 5 years was reported in case of radiotherapy omission, thus underscoring the increasing benefit of radiotherapy with longer follow-up. Based on these studies, the German AGO (Arbeitsgemeinschaft Gynäkologische Onkologie E.V.) and S3 guidelines state that omission of radiotherapy is only an option for patients with limited life expectancy and small, completely resected, hormone receptor-positive tumors [18, 19]. Notably, since the majority of studies investigating the omission of radiotherapy included only patients who had negative lymph node status after SLNB or ALND, pN0 is considered a mandatory criterion in the German guidelines. However, in the CALGB 9353-trial that predated the SLNB era, two thirds of patients did not undergo axillary lymph node dissection [16].

Analyses of the dose distributions from the INSEMA trial in the axillary levels reveal that in 50% of patients, ≥ 85% of the prescribed dose was delivered to axillary level I, and the median dose in the no-SLNB group in level I was 82.7% of the prescribed dose [8]. It should also be noted that most patients in the SOUND and INSEMA trials received endocrine therapy, and adherence to endocrine therapy is known to be higher in clinical trials compared to daily clinical practice [20]. Given that patients with early termination of endocrine therapy generally have a higher risk of local recurrence, it can be assumed that this is particularly true for patients with undetected axillary lymph node involvement after SLNB omission and subsequent omission of radiotherapy [20].

In the INSEMA trial, the primary analysis was conducted in the per protocol population, excluding 252 patients who did not receive radiotherapy. Analyzing this cohort with a follow-up of > 5 years could provide valuable insights into the relevance of adjuvant radiotherapy. Until there are reliable data regarding the omission of SLNB *and* radiotherapy, both de-escalation strategies should not be combined. When discussing de-escalation therapies, it should be noted that only a small proportion of the patients eligible for de-escalation of SLNB (based on the inclusion criteria of SOUND and INSEMA and the current German guidelines) would also be suitable for omission of RT even in the case of pN0.

Also, different from the omission of SLNB, omission of radiotherapy has no effect on the overall quality of life measure, as shown in the randomized PRIME study [21]. Hence, performing SLNB, with its demonstrated negative impact on quality of life, only to fulfill the criteria for omission of adjuvant radiotherapy would thwart the intention of treatment de-escalation for the benefit of improved quality of life.

### Conclusion

- Incidental dose coverage of the axilla during adjuvant radiotherapy likely contributed to the low number of axillary lymph node recurrences despite macroscopic metastases in approximately 10% of patients in the SOUND and INSEMA trials.
- Therefore, after the omission of SLNB, adjuvant breast radiotherapy following lumpectomy is generally recommended and should be considered standard of care.
- Omission of both SLNB and adjuvant radiotherapy is an option for frail patients with limited life expectancy.

## Omitting axillary surgery: implications for partial-breast irradiation

Over 10,000 patients have been enrolled in randomized controlled trials (RCTs) evaluating partial-breast irradiation (PBI). These studies confirm PBI as an oncologically equivalent treatment option compared to whole-breast irradiation (WBI) for patients with early breast cancer, allowing for superior sparing of lung and heart tissue [22].

Based on the existing literature and clinical guidelines, the majority of patients in INSEMA and SOUND would be considered candidates for PBI [19, 19, 23]. Consequently, the question arises as to whether PBI can be offered after SLNB omission.

In the INSEMA trial, PBI was not allowed. In the SOUND trial, 10.8% of patients randomized to the non-SLNB arm received intraoperative electron PBI. However, no subgroup analyses of these patients are available.

Due to smaller fields and often steeper dose gradients [24], the incidental dose to the axilla during PBI is generally lower compared to WBI. Hereby, a large variability depending on the treatment technique, tumor site, and patient anatomy must be expected. Even if the incidental dose in the axillary levels also varies during WBI, the median dose of 85% in axillary level I reported in the INSEMA quality assurance raises concerns about whether PBI provides sufficient coverage in the axilla, especially since clinically undetected lymph node macrometastases occurred in up to 12% of cases.

Although three randomized controlled trials investigating PBI included patients with macroscopic nodal involvement [25–27], the number of patients with pN+ was very low across all trials. Furthermore, it must be expected that a substantial proportion of these patients underwent axillary lymph node dissection (ALND), thus minimizing the relevance of the incidental dose to the axilla. Therefore, both the German and ASTRO guidelines do not recommend PBI for node-positive disease [19, 28].

Meta-analyses of PBI [22] show that regional recurrences, while uncommon, are more frequent in patients treated with PBI compared to WBI (1.0% vs. 0.5%; RR 1.91, 95% CI 1.08–3.39; *P* = 0.03). This difference is likely attributable to variations in the incidental dose. Although this trend was not significant in studies focusing on brachytherapy or external-beam irradiation, the differences could be larger in patients without SLNB due to a higher risk of residual undetected lymph node metastases.

However, neither the SOUND nor the INSEMA trial intentionally treated axillary level I, and in one of the trials, PBI was permitted. Furthermore, careful patient selection, such as limiting inclusion to those with T1, G1–2, HR-positive, HER2-negative tumors might reduce the risk of regional recurrences.

### Conclusion

- Current guidelines include negative pathological lymph node assessment as a selection criterion for PBI.
- In carefully selected cases, a combination of SLNB omission and PBI may be an option after detailed discussion of potential risks and benefits.

## Practical conclusion

INSEMA and SOUND provide practice-changing results for the management of patients with early-stage breast cancer. Considering the excellent oncological results for this population, studying de-escalation strategies to minimize morbidity and maintain quality of life are of utmost importance. However, taking two steps at a time and combining different de-escalation strategies such as PBI and omission of SLNB based on trials that were conducted in parallel may put patients at an increased risk of recurrence. Further analysis of the role of the incidental axillary dose is needed to enhance our understanding and avoid overtreatment. Interdisciplinary discussions of the potential implications of omission of SLNB in individual cases are necessary to optimize the management of our patients.
